# Supplementary material for: Initial Risk Assessment in Patients with Alveolar Echinococcosis—Results from a Retrospective Cohort Study
Source: Pathogens. 2022 May 9;11(5):557. doi: 10.3390/pathogens11050557 (PMC9144025; doi:10.3390/pathogens11050557)
Supplement: Supplementary file 1 [file pathogens-11-00557-s001.zip › pathogens-1601166-supplementary.pdf]

Supplementary Table S1: Results of the univariate analysis

Variable		Clinical outcome				Univariate analysis			
		Cured	Stable with BMZ	Stable without BMZ	Progressive disease	Groupwide comparison		Pairwise comparison for progressive and controlled disease	
						effect size	*p*	*effect size*	*p*
**Demographic variables**									
**Gender**	*N male (%*)*	13 (13.8)	53 (56.4)	16 (17.0)	12 (12.8)	Chi-square-test:		Chi-square-test:	
	N female (%)*	25 (18.8)	77 (57.9)	21 (15.8)	10 (7.5)	$X^2(3) = 2.450$	0.484	$X^2(1) = 0.206$	0.650
**Age at first diagnosis**	*N*	30	130	37	22	ANOVA:		*t*-test:	
	M	**42.5**	**57.6**	**47.8**	**50.7**	$F(3) = 9.319$	**< 0.001**	$t(41) = 0.829$	0.412
	SD	16.4	16.1	19.6	51.5				
	MD	43	58	51.0	18.1				
	Min	15	17	11	23				
	Max	70	89	79	78				
**Occupational disease**	*N yes (%*)*	4 (12.1)	19 (57.6)	4 (12.1)	6 (18.2)	Chi-square-test:		Chi-square-test:	
	N no (%)*	34 (17.9)	111 (58.4)	33 (17.4)	12 (6.3)	$X^2(3) = 5.925$	0.115	$X^2(1) = 1.850$	0.174
**Co-morbidities**									
**Immuno-supression**	*N yes (%*)*	5 (13.9)	20 (55.6)	8 (22.2)	3 (8.3)	Chi-square-test:		Chi-square-test:	
	N no (%)*	33 (17.3)	110 (57.6)	29 (15.2)	19 (9.9)	$X^2(3) = 1.232$	0.745	$X^2(1) = 1.448$	0.229
**Malignancy**	*N yes (%*)*	4 (13.3)	22 (73.3)	2 (6.7)	2 (6.7)	Chi-square-test:		Chi-square-test:	
	*N no (%**)*	34 (17.3)	108 (54.8)	35 (17.8)	20 (10.2)	$X^2(3) = 4.092$	0.252	$X^2(1) = 0.002$	0.961
**Clinical presentation and staging**									
**Clinical symptoms**	*N yes (%**)*	28 (73.7)	89 (69.0)	23 (60.5)	15 (88.2)	Chi-square-test:		Chi-square-test:	
	*N no (%**)*	10 (26.3)	40 (31.0)	15 (39.5)	2 (11.8)	$X^2(3) = 4.804$	0.187	$X^2(1) = 4.416$	**0.036**
**P**	*1: N (%**)*	1 (2.6)	3 (2.4)	1 (2.7)	0 (0)	Kruskal-Wallis-test:		Mann-Whitney-U-test	
	*2: N (%**)*	22 (57.9)	30 (23.6)	22 (59.5)	1 (4.5)	$X^2(3) = 35.536$	**< 0.001**	$U(42) = 30.500$	**< 0.001**
	*3: N (%**)*	6 (15.8)	29 (22.8)	6 (16.2)	3 (13.6)				
	*4: N (%**)*	9 (23.7)	64 (50.4)	8 (21.6)	18 (81.8)				
**N**	*0: N (%**)*	27 (75.0)	76 (61.8)	27 (79.4)	7 (31.8)	Chi-square-test:		Chi-square-test:	
	*1: N (%**)*	9 (25.0)	47 (38.2)	7 (20.6)	15 (68.2)	$X^2(3) = 15.487$	**0.001**	$X^2(1) = 15.465$	**< 0.001**

**M**	0: N (%**)	37 (97.4)	110 (88.0)	35 (94.6)	18 (81.8)	Chi-square-test:		Chi-square-test:	
	1: N (%**)	1 (2.6)	15 (12.0)	2 (5.4)	4 (18.2)	$X^2(3) = 5.393$	0.145	$X^2(1) = 0.170$	0.345
**Staging**	Stage I: N (%**)	1 (2.6%)	3 (2.3%)	1 (2.6%)	0 (0%)	Chi-square-test:		Chi-square-test:	
	Stage II: N (%**)	19 (48.7%)	23 (17.4%)	22 (56.4%)	0 (0%)				
	Stage IIIa: N (%**)	5 (12.8%)	21 (15.9%)	5 (12.8%)	1 (4.5%)	$X^2(12) = 56.313$	**< 0.001**	$X^2(8) = 44.562$	**< 0.001**
	Stage IIIb: N (%**)	7 (17.9%)	44 (33.3%)	3 (7.7%)	5 (22.7%)				
	Stage IV: N (%**)	7 (17.9%)	41 (31.1%)	8 (20.5%)	16 (72.7%)				
**Curative resection possible**	N yes (%*)	38 (36.1)	38 (36.1)	25 (24.9)	3 (2.9)	Chi-square-test:		Chi-square-test:	
	N no (%*)	0.0 (0)	91 (74.6)	12 (9.8)	19 (15.6)	$X^2(3) = 75.021$	**< 0.001**	$X^2(1) = 10.714$	**0.002**
**Laboratory results at first presentation**									
**ALT** in IU/l	*N*	31	113	30	19	ANOVA:		*t*-test	
	M	**51.6**	**59.8**	**52.2**	**90.4**				
	SD	50.7	104.0	54.1	192.3				
	MD	31.0	28.0	28.0	23.0	$F(3) = 0.666$	0.574	$t(35) = -1.020$	0.315
	Min	13.0	11.0	11.0	13.0				
	Max	239.0	796.0	264	850.0				
**γGT** in IU/l	*N*	31	113	30	19	ANOVA:		*t*-test	
	M	**70.5**	**122.9**	**112.8**	**183.7**				
	SD	95.4	161.3	297.6	289.0				
	MD	36.0	60.0	31.0	82.0	$F(3) = 1.348$	0.260	$t(35) = -1.916$	0.064
	Min	10.0	4.0	12.0	13.0				
	Max	490.0	902.0	1,606	1,117.0				
**AP** in IU/l	*N*	24	98	23	17	ANOVA:		*t*-test	
	M	**86.8**	**126.5**	**124.0**	**224.4**				
	SD	56.8	123.8	184.2	402.5				
	MD	70.5	88.5	70.0	80.0	$F(3) = 2.129$	0.099	$t(29) = -1.458$	0.156
	Min	34.0	30.0	36.0	27.0				
	Max	306.0	880.0	812.0	1,720.0				
**Bilirubin** in mmol/l	*N*	24	84	20	17	ANOVA:		*t*-test	
	M	**8.4**	**18.6**	**13.4**	**26.8**				
	SD	3.0	33.5	10.1	75.5	$F(3) = 0.969$	0.409	$t(27) = -0.734$	0.469
	MD	8.3	8.0	11.0	9.0				

	Min	2.0	2.0	4.0	4.0				
	Max	17.0	211.0	46.0	319.0				
**CRP** in mg/l	*N*	19	61	19	13	ANOVA:		*t*-test	
	M	**12.1**	**13.9**	**7.0**	**17.3**	*F*(3) = 0.458	0.712	*t*(22) = -0.704	0.489
	SD	29.7	28.4	17.2	27.2				
	MD	3.3	3.2	2.7	6.3				
	Min	0.4	0.4	0.3	1.5				
	Max	125.0	171.4	76.1	100.0				
**IgE** in IU/ml	*N*	38	129	37	22	ANOVA:		*t*-test	
	M	**821.3**	**1,123.0**	**210.4**	**1,833.9**	*F*(3) = 2.713	**0.046**	*t*(41) = -3.026	**0.004**
	SD	2,344.7	2,459.6	497.7	2640.5				
	MD	149.5	237.0	36.0	513.5				
	Min	1.0	1.0	1.0	8.0				
	Max	13,400.0	16,637.0	2,469.0	8,987.0				
**Ech. IHA** 1:	*N*	36	104	37	21	Kruskal-Wallis-test:		Mann-Whitney-U-test	
	M	1,008.0	712.0	73.7	1,076.6	X^2(3) = 23.907	**< 0.001**	*U*(42) = 75.000	**< 0.001**
	SD	5,448.7	3,299.1	334.8	1,944.3				
	MD	**16.0**	**48.0**	**0.0**	**100.0**				
	Min	0.0	0.0	0.0	0.0				
	Max	32,768	32,768	2,024	8,192				
**Ech. EIA IgG** in U/l[1]	*N*	4	78	8	12	ANOVA:		*t*-test	
	M	**91.6**	**75.6**	**32.1**	**128.6**	*F*(3) = 4.020	**0.010**	*t*(17) = -3.021	**0.008**
	SD	149.4	57.3	38.4	77.4				
	MD	27.0	65.6	22.8	100.0				
	Min	0	0	0	49				
	Max	312.0	329.0	108	299				
**Em2+**	N negative (%**)	8 (21.6)	19 (15.0)	21 (58.3)	1 (4.5)	Chi-square-test:		Chi-square-test:	
	N positive (%**)	29 (78.4)	108 (85.0)	15 (41.7)	21 (95.5)	X^2(3) = 35.188	**< 0.001**	X^2(1) = 22.051	**< 0.001**
**Imaging at first presentation (US)**									
**Largest diameter** in mm	*N*	23	103	27	18	ANOVA:		*t*-test	
	M	**71.5**	**64.0**	**38.0**	**107.4**	*F*(3) = 11.614	**< 0.001**	*t*(17) = -6.468	**< 0.001**
	SD	48.6	35.3	37.2	47.8				
	MD	61.0	62.0	22.0	101.5				
	Min	12	7	10	34				

	Max	177	181	177	222				
**Number of lesions**	*N*	37	108	36	20	ANOVA:		*t*-test	
	M	**1.14**	**2.7**	**1.72**	**1.55**				
	SD	1.3	2.8	2.0	0.95	*F*(3) = 4.997	**0.002**	*t*(39) = -1.761	0.086
	MD	1.0	2.0	1.0	1.0				
	Min	0	1	1	1				
	Max	5	20	20	4				
**Imaging during course of disease (US/CT/MRI/ PET-scan)**									
**Morphology (EMUC-US)** N (%*)[2]	hailstorm	0 (0)	49 (79.0)	6 (9.7)	7 (11.3)	Chi-square-test:		Chi-square-test:	
	haemangioma-like	0 (0)	9 (69.2)	2 (15.4)	2 (15.4)	X^2(21) = 148.512	**< 0.001**	X^2(7) = 19.231	**0.007**
	pseudocystic	0 (0)	10 (58.8)	0 (0)	7 (41.2)				
	ossification	0 (0)	1 (50.0)	1 (50.0)	0 (0)				
	metastasis-like	0 (0)	14 (60.9)	9 (39.1)	0 (0)				
**Cholestasis**	*N yes (%**)*	3 (7.9)	24 (18.6)	6 (16.2)	7 (43.8)	Chi-square-test:		Chi-square-test:	
	*N no (%**)*	35 (92.1)	105 (81.4)	31 (83.8)	9 (56.3)	X^2(3) = 9.846	**0.020**	X^2(1) = 11.331	**0.001**
**Portal vein involvement**	*N yes (%**)*	4 (10.5)	25 (19.4)	2 (5.2)	10 (62.5)	Chi-square-test:		Chi-square-test:	
	*N no (%**)*	34 (89.5)	104 (80.6)	35 (94.6)	6 (37.5)	X^2(3) = 26.269	**< 0.001**	X^2(1) = 17.986	**< 0.001**
**V. cava involvement**	*N yes (%**)*	3 (7.9)	29 (22.5)	3 (8.1)	6 (37.5)	Chi-square-test:		Chi-square-test:	
	*N no (%**)*	35 (92.1)	199 (77.5)	34 (91.9)	10 (62.5)	X^2(3) = 10.608	**0.014**	X^2(1) = 6.345	**0.012**
**Treatment**									
**Unplanned treatment interruption**	*N yes (%**)*	7 (18.9)	12 (9.2)	7 (21.2)	8 (36.4)	Chi-square-test:		Chi-square-test:	
	*N no (%**)*	30 (81.1)	118 (90.8)	26 (78.8)	14 (63.6)	X^2(3) = 12.481	**0.006**	X^2(1) = 0.005	0.945
**Mean albendazole blood level**	*N*	36	126	34	20	ANOVA:		*t*-test	
	M	**0.70**	**0.37**	**0.48**	**0.43**	*F*(3) = 3.082	**< 0.001**	*t*(36) = -0.157	0.876
	SD	0.46	0.39	0.38	0.41				
	MD	0.68	0.29	0.51	0.43				
	Min	0.0	0.0	0.0	0.0				
	Max	1.75	1.40	1.50	1.29				

* percentage of patients from all patients with/without respective variable ** percentage of patients from all patients with respective outcome

1 = the serological test for EIA IgG was introduced in 2018. Additionally, we were able to examine several samples from 2012 to 2018 retrospectively, however, not for all patients in our cohort. That explains the proportion of missing values.

2 = The morphological classification of AE lesions according to EMUC-US was introduced during the study period. Therefore, morphology descriptions from the last visit were evaluated. Hence, cured patients who underwent surgery did no longer have any AE lesions.
